# The Endocannabinoid System Differentially Regulates Escape Behavior in Mice

**DOI:** 10.3389/fnbeh.2017.00201

**Published:** 2017-10-20

**Authors:** Andreas J. Genewsky, Carsten T. Wotjak

**Affiliations:** RG Neuronal Plasticity, Department of Stress Neurobiology and Neurogenetics, Max Planck Institute of Psychiatry, Munich, Germany

**Keywords:** endocannabinoid system, active fear, passive fear, behavioral inhibition, conditional knock-out, avoidance

## Abstract

Among the *hardwired* behaviors, fear or survival responses certainly belong to the most evolutionary conserved ones. However, higher animals possess the ability to adapt to certain environments (e.g., novel foraging grounds), and, therefore, those responses need to be plastic. Previous studies revealed a cell-type specific role of the endocannabinoid system in novelty fear, conditioned fear and active vs. passive avoidance in a shuttle box paradigm. In this study we aim to investigate, whether knocking-out the cannabinoid receptor type-1 (CB1) on cortical glutamatergic (Glu-CB1^−/−^) or GABAergic (GABA-CB1^−/−^) neurons differentially affects the level of behavioral inhibition, which could ultimately lead to differences in escape behavior. In this context, we developed a novel behavioral paradigm, the *Moving Wall Box* (MWB). Using the MWB task we could show that Glu-CB1^−/−^ mice have higher levels of behavioral inhibition over the course of repeated testing. GABA-CB1^−/−^ mice, in contrast, showed significantly lower levels of behavioral inhibition compared to wild-type controls and more escape behavior. These changes in behavioral inhibition and escape behavior cannot be explained by altered levels of arousal, as repeated startle measurements revealed general habituation irrespective of the line and genotype of the animals. Taken together, we could show that CB1 on cortical glutamatergic terminals is important for the acquisition of active avoidance, as the absence of CB1 on these neurons creates a bias toward inhibitory avoidance. This is the case in situations without punishment such as electric footshocks. On the contrary CB1 receptors on GABAergic neurons mediate the acquisition of passive avoidance, as the absence of CB1 on those neurons establishes a strong bias toward escape behavior.

## 1. Introduction

The endocannabinoid (eCB) system is a phylogenetically ancient neuromodulatory system, and genes encoding for the cannabinoid receptors CB1 and/or CB2 can be found most likely in all chordates (Elphick, 2012). Its endogenous ligands (endocannabinoides) are synthesized and released on demand from postsynaptic sites. They travel to presynaptically localized CB1 receptors, where they cause a decrease in transmitter release in an auto- and heterosynaptic manner. This feedback mechanism has been found to function as an important regulator in the balance of excitatory and inhibitory neurotransmission (Kano et al., 2003) and, hence, is a major system which mediates adaptation at the synaptic level. As evolutionary conserved as the eCB system itself, are also behavioral responses (i.e., stress and fear responses) which allow an individual to remove itself from dangerous situations. Higher vertebrates, like mammals, have a complex behavioral repertoire and are able to adapt the expression of defensive responses to certain environmental cues. This allows hemerophile species, like mice and rats, to explore foraging grounds and habitats which are inaccessible to other species. The role of the eCB system in the regulation of emotion, stress and fear responses has been implicated numerous times (Wotjak, 2005; Lutz, 2009; Hill et al., 2010; Riebe et al., 2012; Ruehle et al., 2012) which is substantiated by the reports of euphoria upon the recreational drug use of marijuana and cannabis extracts. Special attention received the bimodal role of eCB signaling on glutamatergic vs. GABAergic neurons in the adoption of active and passive fear coping strategies in shuttle box training using electric footshocks (Metna-Laurent et al., 2012) in mice: whereas the cell-type specific knock-out of CB1 on glutamatergic cortical neurons (Glu-CB1^−/−^) increased performance in a passive avoidance task and impaired active avoidance, the opposite was observed when CB1 was absent on GABAergic (GABA-CB1^−/−^) forebrain neurons (decreased freezing, increased performance in an active avoidance task, impaired passive avoidance). A similar differential involvement of CB1 on glutamatergic vs. GABAergic neurons has been observed also in conditioned fear (Dubreucq et al., 2012; Llorente-Berzal et al., 2015), novel object exploration (single-housed animals) (Lafenêtre et al., 2009) and fasting-induced food intake (Bellocchio et al., 2010) (*for review see* Lutz et al., 2015). It has been suggested, that the eCB system is activated on demand (Di Marzo et al., 1998) upon the substantial activation of the synapse, and the effects of Glu-CB1^−/−^ and GABA-CB1^−/−^ might therefore only precipitate after a strong stimulus combined with sufficient incubation time. Experimental paradigms which involve social isolation (single-housed animals), repeated physical punishments or painful stimuli (e.g., electric footshock) may activate the eCB system beforehand. In consequence, the temporal dynamics of the eCB system during repeated testing cannot be assessed anymore. More importantly, the observed behavioral differences in conditional CB1 knock-out animals may relate to altered processing of the unconditioned stimulus, rather than to cognitive processes. Such a scenario is supported by the implication of the eCB system in pain perception (Woodhams et al., 2017). Thus, for the study of its role in modulating a particular behavior, the use of experimental paradigms which do not involve painful stimuli and offer repeated testing are of considerable importance.

Here we describe a novel behavioral assay—the *Moving Wall Box* (MWB) task, which allows the repeated assessment of fear coping strategies without the need of preceding aversive conditioning or any other form of operant training involving footshocks, food or water deprivation. Using the MWB task we demonstrate the time-dependent involvement of the eCB system in the generation of active vs. passive coping strategies depending on the neuronal cell-type affected.

## 2. Materials and methods

### 2.1. Animals

We used adult (4–8 months), male, group-housed CB1^f/f;NEX-Cre^ (Monory et al., 2006), henceforward called Glu-CB1^−/−^)(*N* = 9) and CB1^f/f;Dlx5/6-Cre^ (Monory et al., 2006), henceforward called GABA-CB1^−/−^)(*N* = 9) and their corresponding wild-type litter mate controls, Glu-CB1^+/+^(*N* = 9) and GABA-CB1^+/+^(*N* = 10). All animals were bred in the animal facilities of the Max Planck Institute of Biochemistry, Martinsried, Germany. The animals were group-housed (2–4 animals per cage) under standard housing conditions: 12 h/12 h inverted light-dark cycle (light off at 8 AM), temperature 24°C, food and water *ad libitum*. Experimental procedures were approved (AZ 44-09) by the State of Bavaria (Regierung von Oberbayern, Munich, Germany). Animal husbandry and experiments were performed in strict compliance with the European Economic Community (EEC) recommendations for the care and use of laboratory animals (2010/63/EU). On the basis of prior power analysis, we have kept the number of animals at the absolute minimum, sufficient to reveal significant group differences.

### 2.2. Apparatus and behavioral paradigm

**Figure 1:**
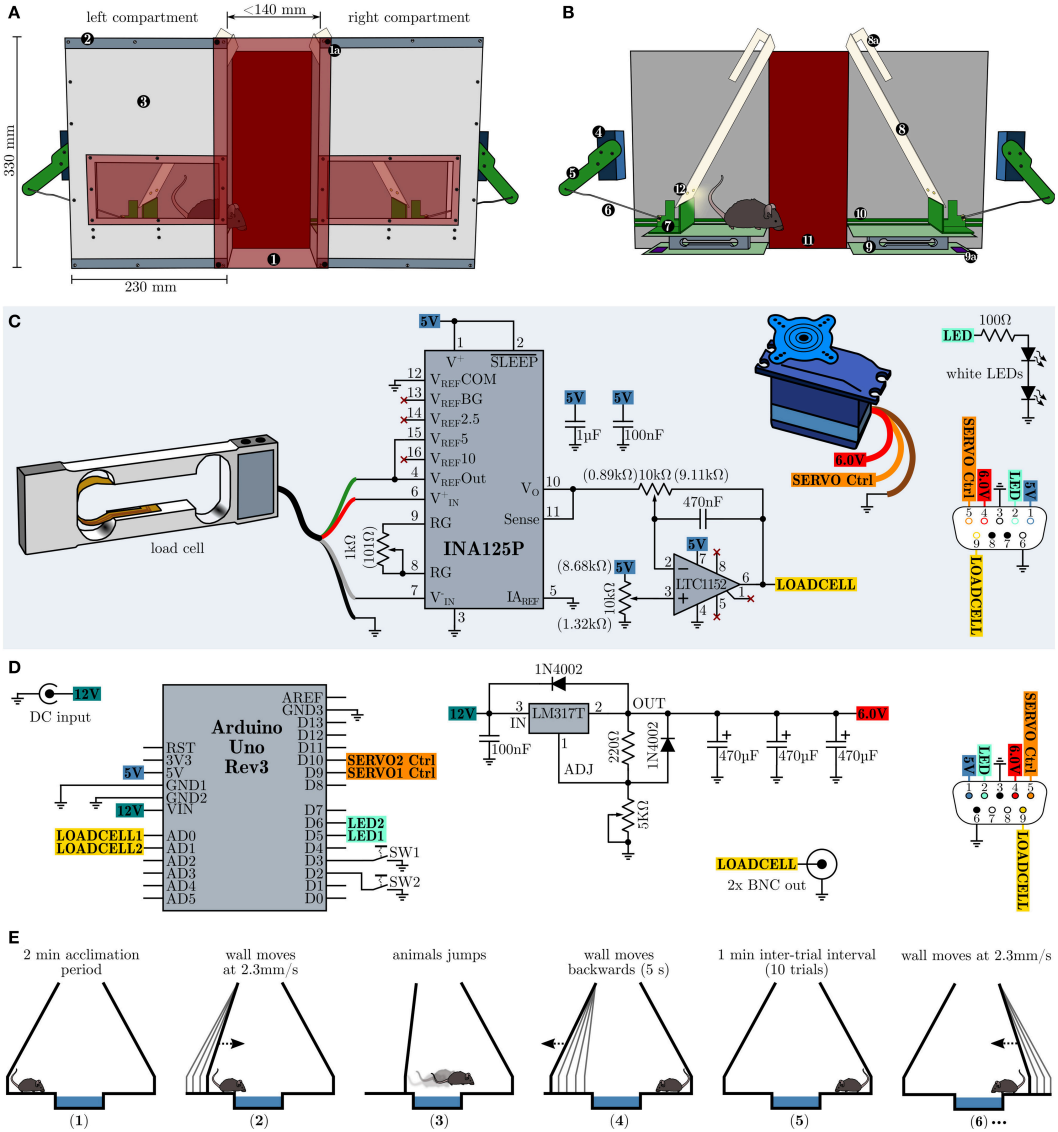
Design, schematics and operational procedure of the moving wall box (MWB). **(A)** Overview of the MWB. (1) Red transparent front cover which is equipped with four strong neodymium magnets at its corners (1a) and allows to adjust the distance between the left and the right compartment via metal bands (2). The front cover of the compartments (3) is equipped with a red transparent window, allowing to observe the animal at all times. **(B)** Inner workings of the MWB. Servo motor (4); articulated joint, consisting of an arm (5) and a rod (6); sliding carriage (7) supporting the moving wall (8), which itself is equipped with slotted mounts (8a); balance (9), consisting of two opposite plates and the load cell signal conditioning circuit (9a); rails (10) for the sliding carriage; red transparent back cover (11); two white LEDs per wall (12). **(C)** Schematic depiction of the circuitry of one compartment. Potentiometer settings which gave best results are given in brackets. **(D)** MWB controller circuity. **(E)** Schematic representation of the MWB task procedure. (1) acclimatization for 2 min; (2) the wall of the compartment in which the mouse resides starts to move with constant speed of 2.3 mm/s, maximal for 60 s (138 mm); (3) the wall stops to move once the animal has shuttled to the opposite compartment; (4 and 5) during a 1 min inter-trial interval the first wall move quickly back to its default position; (6) subsequently the wall of the compartment in which the mouse resides starts to move and the second trial has started. This cycle is repeated 10× per session.

In order to repeatedly assess the development of behavioral inhibition in an emotional challenging situation without footshocks, food or water deprivation, we devised a novel testing strategy, henceforward called the Moving Wall Box (MWB) task. In short, during the MWB task a mouse is repeatedly forced to jump over a small ice-filled box (10 trials, 1 min inter-trial intervals ITI), by slowly moving walls (2.3 mm/s, over 60 s), whereby the presence of the animal is automatically sensed via balances and analyzed by a microcontroller board which in turn controls the movements of the walls. The behavioral readouts are (1) the latency to reach the other compartment (high levels of behavioral inhibition lead to high latencies) and (2) the number of inter-trial shuttles per trial (low level of behavioral inhibition lead to high levels of shuttles during the ITI). The MWB, depicted in Figure 1A, consists of two separate compartments, connected via two red transparent acrylic glass plates (W160 × H340 × D4 mm) which are outfitted with strong neodymium magnets at their corners. The magnets in turn allow to adjust the space between the two compartments as they can be attached along the top and bottom metal bands on each compartment. The compartments hold a window at their at their front panels for unrestricted visual access to the animal inside, at all times. Each compartment possesses one servo motor (Bluebird or Turnigy 620DMG+HS) which is connected via an articulated joint (Figure 1B), consisting of an arm (8 cm), mounted to the servo motor and a rod (14 cm) connecting to the sliding carriage. The sliding carriage can freely travel along the entire width of one compartment, while being supported by a pair of rails mounted at the inner faces of the rear and front panels. Supported by the sliding carriage is the eponymous moving wall, which is further hanging via two long slotted mounts from a short rod (mounted upper-midways between the rear and front panel of each compartment). Once the sliding carriage is pushed toward the mid, the resting wall simply moves up- and forwards. The presence of the mouse is sensed via load cell units (details *see below*, surface W160 × D100 mm) and its output is amplified and filtered with a dedicated amplifier circuit (Figure 1C) and fed toward a microcontroller board with little auxiliary circuitry (Figure 1D) which in turn controls the servo motors as well as a pair of LED per wall which illuminate the active compartment.

The load cell (Tedea Huntleigh 1004-00.3-JW00-RS, 0.3 kg, Figure 1C) is connected to an INA125P instrumentation amplifier (Texas Instruments) which is configured to provide a gain of 600 ×. The output of the instrumentation amplifier is further fed toward a high performance Rail-to-Rail I/O zero-drift operational amplifier LTC1152 (Linear Technology) which is configured as an inverting stage with a gain of 10 × and includes a low-pass filter (−3 dB at 35 Hz). The load cell circuit, the servo motor and the white LEDs (e.g., Cree XLamp XM-L2 or Osram Oslon SSL 80) of one compartment are connected via a female D-sub-miniature 9-pin (DB9) receptacle and the wiring diagram is shown in Figure 1C at the lower right side. The MWB controller (Figure 1D) is powered via an external stabilized 12 V power supply (>750 mA) and houses an Arduino Uno Rev3 microcontroller prototyping platform which interfaces to a PC via USB, running the MWB graphical user interface, and controls the movements of the servo motors. The servo motor voltage supply is implemented using the adjustable voltage regulator LM317T (TO-220) set to provide stable 6.0 V voltage supply. The LM317T should be protected from overheat using a standard TO-220 heat sink with a thermal resistance of 7.5–10°C/W. The outputs of the MWB controller interfaces with the compartments via male DB9 connectors, whose wiring diagram is depicted in Figure 1D at the lower right side. In addition the MWB controller provides the filtered output of the load cell circuits via two BNC connectors. This is useful if, at later stages, *in vivo* electrophysiological recordings are attempted in order to align neural responses to the time of jump. However, given the low frequency response of the low-pass filter, one should consider to tap the INA125P output directly and route it via the remaining free pins of the DB9 connectors, in order to obtain fast load cell voltage outputs. All circuits can be built using perfboard, but in order to interface with the Arduino Uno directly, specific Arduino Proto Shields (e.g., Adafruit PID: 2077 or SparkFun DEV-07914) have been found very useful. The total building costs of one MWB unit are less than 400 $. Compared to other commercially available behavioral apparatuses which allow the assessment of active vs. passive fear coping behaviors (e.g., shuttle-box), the asset costs of our proposed design are low and the complexity of the electronic circuit can be easily mastered by an electronics novice. We deliberately omitted detailed dimensions of all mechanical parts as we feel that these specifications are heavily dependent on the materials in use and unnecessarily hinder a successful copy of our design. However, more detailed building instructions are available on request. Further, the concept of the behavioral apparatus (a mechanism which gently forces an animal to overcome an obstacle combined with a presence sensing circuit) can be realized in various different and possibly even more elegant ways (e.g., optical detection). The behavioral paradigm using the MWB is simple and straightforward (Figure 1E). Before each session (consisting of 10 trials) a small container (W14 × H3 × D10 cm) is filled with crushed ice and placed in between the two compartments. In addition the apparatus should be wiped with water and detergent. The mouse is placed at compartment A and left 2 min to acclimatize. Subsequently the left wall starts to move slowly and forces the animals to walk/jump over the ice. The time from the onset of the movement of the wall to the time when the animal is reaching the other compartment is the latency. During the inter-trial interval (ITI) of 1 min, the left wall is moving back to the default position, and the animal can perform a certain number of inter-trial shuttles between the compartments. The compartment in which the animal resides after ITI expiration, will become active next.

### 2.3. Acoustic startle reflex measurements

In order to assess the animals general arousal level in a non-invasive manner with minimal stress, we have employed acoustic startle measurements using a modified version of a commercially available startle apparatus (SR-LAB™, San Diego Instruments), which allowed unrestrained movements of the animals. The modification involved the replacement of the restraining startle chamber with a customized version, based on a Makrolon type II cage (27 × 16 × 12 cm) where all walls were cut to a height of 5 cm and the floor plate was allowed to translate the animals movements by placing long 3 mm wide slits along the bottom edges (leaving 1 cm fillets at the corners). A Ø5 cm piezoelectric transducer was glued (Pattex Stabilit Express) to the center from outside and its output was fed via a 6.3 mm audio jack toward the startle apparatus input. In addition we have placed four black walls (3 mm, PROTEX, rigid PFC foam plate) 5 mm above the floor plate, inside this modified cage, to force the animal to reside roughly above the sensor. This resulted in sufficient space (W15 × H16 × D9 cm) for the animals to move freely while receiving startle pulses. Rubber feet at the corners, isolated the startle chamber from unwanted vibrational signals. To account for the animals inter-individual difference in startle responsivity, we have first (7 days before MWB task) determined an individual input-output (I/O) response using a startle protocol which involved the display of 15 white noise startle pulses (50 ms) per sound pressure level (SPL) ranging from 70 to 120 dB in increments of 10 dB with variable inter-pulse intervals of 7 s to 15 s in a pseudo-randomized manner. In addition each animal received 10 startle pulses (5 × 70 dB + 5 × 100 dB) before the I/O session (acclimatization), whose responses have been discarded, giving a total number of 100 startle pulses. Based on these individual I/O curves, we have selected the SPL which was closest to the half-maximal response (SPL_50_). After each MWB session on the same day, the animals have been subjected to a startle session which involved 50 startle pulse at the individual SPL_50_ with variable inter-pulse intervals of 7–15 s.

### 2.4. Software design

The cross-platform software to write and upload the Arduino code used in this study is freely available online^1^. In addition all files (Arduino firmware and Python GUI for controlling the MWB) are available online^2^ or on request.

### 2.5. Statistical analysis

All data are presented as mean values ± standard error (SEM). Statistical analysis has been performed using GraphPad Prism 5.03. Two-way analysis of variance (ANOVA for repeated measures) was followed by *Bonferroni post-hoc* analysis. A *p* < 0.05 was considered statistically significant.

## 3. Results

**Figure 2:**
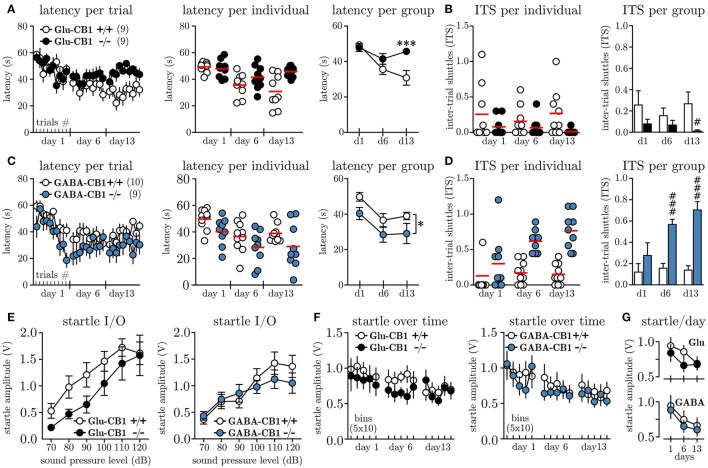
Conditional knock-out of CB1 on glutamatergic vs. GABAergic neurons differentially affects escape behavior. **(A)** Latencies to shuttle to the opposite compartment during the MWB task, given per trial (*left panel*), per individual and day (*mid panel*) and per group and day (*right panel*) for Glu-CB1^+/+^(*N* = 9) and Glu-CB1^−/−^(*N* = 9). **(B)** Inter-trial shuttles per individual and day (*left panel*) and per group and day (*right panel*) for Glu-CB1^+/+^ and Glu-CB1^−/−^. **(C)** Latencies to shuttle to the opposite compartment during the MWB task, given per trial (*left panel*), per individual and day (*mid panel*) and per group and day (*right panel*) for GABA-CB1^+/+^(*N* = 10) and GABA-CB1^−/−^(*N* = 9). **(D)** Inter-trial shuttles per individual and day (*left panel*) and per group and day (*right panel*) for GABA-CB1^+/+^ and GABA-CB1^−/−^. **(E)** Startle input-output (I/O) curves for Glu-CB1 and GABA-CB1 animals. **(F)** Startle response at SPL_50_ development over time depicted in bins of 10 trials. **(G)** Same Same data as in (F) but shown as average startle amplitude at SPL_50_ per day for the individual groups for (*upper panel*) Glu-CB1^+/+^ (empty circles) and Glu-CB1^−/−^ (filled circles) animals and (*lower panel*) GABA-CB1^+/+^ (empty circles) and GABA-CB1^−/−^ (filled circles) animals. Asterisks indicate significance values obtained by repeated measures two-way ANOVA followed by Bonferroni *post-hoc* test: ^*^ *p* < 0.05, ^***^p < 0.001. Hashes indicate significance values obtained by Mann-Whitney *U*-tests: # *p* < 0.05, ### *p* < 0.001. All values are given as mean ± SEM. Red bars in individual data represent the mean value.

In order to investigate whether the cell-type specific knock-out of the CB1 receptor on glutamatergic vs. GABAergic neurons affects the level of behavioral inhibition, we subjected Glu-CB1^−/−^, GABA-CB1^−/−^ and their respective litter mate controls (Glu-CB1^+/+^, GABA-CB1^+/+^) to the MWB task. Once the walls start to move, the animals can stay for maximally 60 s within the initial compartment before they are forced to enter the ice and ultimately reach the opposite compartment. Figure 2A depicts the latencies to reach the opposite compartment for Glu-CB1 animals per trial. Within the first session, all animals adapt to the task, as seen by the decreasing latencies from the first to the last trial. These within-session dynamics could not be observed on day 6, nor on day 13. The individual data per day (Figure 2A, mid panel), visualizes the increasing variation among Glu-CB1^+/+^ mice with time, while the knock-out mice show a very robust response. The grouped data per day (Figure 2A, right panel) reveals the time-dependent development of a profound group difference, between Glu-CB1^+/+^ and Glu-CB1^−/−^ animals with significant group × time interaction [*F*_(2, 34)_ = 8.66, *p* = 0.0009]. The difference was strongest on day 13 when Glu-CB1^+/+^ mice spent on average 30.3 ± 3.7 s before they shuttled while Glu-CB1^−/−^ needed 45.7 ± 1.3 s. In other words, while the wild type animals controlled the situation and responded preemptively before the wall was pushing them (≈ 6.8 cm), the knock-out remained until there was only ≈ 3.3 cm between the wall and the ice. The high latencies were accompanied by a low disposition to show active escape attempts reflected by decreased number of inter-trial shuttles (ITS, Figure 2B, left panel). Statistical analysis between groups per day (non-parametric, two-tailed Mann-Whitney *U*-test) revealed a significant lower ITS values (Figure 2B, left panel) for Glu-CB1^−/−^ mice at day 13 (0.01 ± 0.01 ITS vs. 0.27 ± 0.10 ITS, *U*_*n*1 = 129, *n*2 = 61_ = 16.00, *p* = 0.0094). The percentage of Glu-CB1^+/+^ animals which performed one or more ITS slightly increased from day 1 (50 %) to day 13 (70 %) but the values for Glu-CB1^−/−^ decreased from day 1 (30 %) to day 13 (0 %). The analysis of the contingency tables using Fisher's exact test revealed a significant difference between Glu-CB1^+/+^ and Glu-CB1^−/−^ for ITS values on day 13 (*p* = 0.0198).

Similar to the Glu-CB1, also GABA-CB1 animals showed an initial adaptation to the MWB task within the first session (Figure 2C, left panel), whereby especially GABA-CB1^−/−^ mice seemed to show quicker preemptive responses. These within-session dynamics could not be observed in subsequent sessions. The variance among the groups was similar throughout the experiment, except that GABA-CB1^+/+^ mice showed less variable responses on day 13 (Figure 2C, left panel). Looking at the average latencies per group and day revealed an overall lower latency for GABA-CB1^−/−^ mice [*F*_(1, 17)_ = 4.6, *p* = 0.0466]. Whereas GABA-CB1^−/−^ animals already transitioned to the other compartment after 29.1 ± 5.7 s (≈ 7.1 cm between wall and ice), GABA-CB1^+/+^ needed 38.1 ± 2.0 s, which corresponds to ≈ 5.0 cm before the wall would have pushed them.

In the first session the number of ITS for both groups was comparable, but starting at day 6, both groups separated almost completely (Figure 2D, left panel). Statistical analysis between groups per day (non-parametric, two-tailed Mann-Whitney *U*-test) revealed on average a significant higher number of ITS per trial for GABA-CB1^−/−^ on day 6 (0.56 ± 0.05 ITS vs. 0.17 ± 0.05 ITS, *U*_n1 = 58, n2 = 132_ = 3.00, *p* = 0.0006), and the difference was even more pronounced on day 13 (0.69 ± 0.05 ITS vs. 0.17 ± 0.05 ITS, *U*_n1 = 58, n2 = 132_ = 3.00, *p* = 0.0006). Looking at the percentage of GABA-CB1^+/+^ animals which performed at least one ITS per trial, a clear increase from day 1 (20 %) to day 13 (70 %) was noted. The same was the case for GABA-CB1^−/−^ animals (d1: 67 %; d13: 100 %) with no difference between genotypes (*p* = 0.2105; Fisher's exact).

In order to monitor the arousal level of Glu-CB1 and GABA-CB1 animals throughout the experiment we have applied acoustic startle measurements along the MWB task. To account for inter-individual differences in startle response, we first subjected all animals 7 days before the first MWB session to a startle input-output (I/O) protocol, which allowed us to determine the sound pressure level which yielded a half-maximal response (SPL_50_) for each individual. The results of the startle I/O experiments for Glu-CB1 mice are shown in Figure 2E, (left panel); no significant group difference was found [repeated measures two-way ANOVA, group *F*_(1, 16)_ = 2.48, *p* = 0.1352]. For GABA-CB1 mice, the results of the startle I/O experiment are shown in Figure 2E (right panel), and also here no group difference was observed [repeated measures two-way ANOVA, group *F*_(1, 17)_ = 0.28, *p* = 0.6068]. Based on the I/O measurements the average SPL_50_ were determined for Glu-CB1 (WT 85.6 ± 2.4 dB, KO 92.2 ± 3.2 dB) and GABA-CB1 (WT 86.0 ± 2.2 dB, KO 83.3 ± 2.4 dB) mice. A non-parametric Kruskal-Wallis analysis revealed no significant difference among the groups (*p*≥0.127). These individual SPL_50_ values were used to assess the general arousal on each experimental day after each MWB session. Figure 2F shows the startle responses (one bin represents the average of 10 startle responses) of Glu-CB1 and GABA-CB1 animals. The habituation, reflected by decreasing startle amplitudes over time per testing day (Figure 2G), was revealed by two-way ANOVA [Glu-CB1 *F*_(2, 32)_ = 6.76, *p* = 0.0036; GABA-CB1 *F*_(2, 34)_ = 15.47, *p* < 0.0001], with no effect of genotype (Glu-CB1 *F*_1, 16_ = 0.68, *p* = 0.4212; GABA-CB1 *F*_1, 17_ = 0.21, *p* = 0.6514) or genotype × day interaction [Glu-CB1 *F*_(2, 32)_ = 1.23, *p* = 0.3062; GABA-CB1 *F*_(2, 34)_ = 0.07, *p* = 0.9341].

## 4. Discussion

The prior activation of the eCB system with noxious stimuli, water restriction, food deprivation or social isolation (single-housed animals) is a confounding factor in many experiments aiming to investigate the involvement of endocannabinoid signaling in a certain behavior. Consequently different behavioral assays need to be employed to overcome this limitation. In this context, we have developed the Moving Wall Box task, which allows the assessment of escape behavior in mice. The MWB task does not require painful aversive stimuli (e.g., electric footshock) nor is any other manipulation necessary to motivate the animals to participate in the task (e.g., food or water deprivation). The MWB task is devoid of lengthy training sessions and allows the animal to control the situation without negatively affecting the obtained results. Most importantly the MWB task can be easily repeated which is in steep contrast to many other behavioral paradigms which are based on, e.g., the intrinsic exploratory drive of the animals. Further, the MWB offers the possibility to conduct simultaneous *in vivo* electrophysiological recordings, which could be later aligned to the behavioral responses (escapes). Therefore the MWB task fosters the study of activity patterns in, e.g., optogenetically identified neurons with respect to escape responses in a highly controlled setting. To our knowledge there is no other available compatible behavioral paradigm.

Using this new behavioral paradigm, we could demonstrate that the level of behavioral inhibition, i.e., the balance between active and passive fear coping strategies, is differentially affected by the absence of CB1 on glutamatergic vs. GABAergic neurons in a gradual, time-dependent manner. While GABA-CB1^−/−^ animals show a strong disposition to actively evade impeding danger, Glu-CB1^−/−^ animals are behaviorally inhibited and behave much more passively. The latencies to escape could possibly depend on the general arousal levels of these animals, and their genetic disposition might render them differentially sensitive to stress. The parallel assessment of startle amplitudes over the course of the experiment, however, showed no sensitization of a specific group, but revealed an overall tendency to habituate to the experimental procedures, as the startle amplitudes declined over the 3 testing days. This excludes a general deficit of Glu-CB1^−/−^ in long-term habituation of defensive responses.

The role of CB1 in the regulation of coping styles has been highlighted by Metna-Laurent et al. (2012), however in this study the authors observed different levels of freezing upon a previously aversively conditioned tone. The noxious quality of the unconditioned stimulus (US) activated the eCB beforehand, and the authors observed a bimodal modulation of fear coping strategies already at the first tone-fear memory recall session. Another confounding factor in this behavioral task is the high degree of freedom to display different active behaviors like sniffing, rearing, digging, which all have to be scored by an experienced experimenter, blind to the conditions. In the MWB, the analysis is based on two simple parameters and the animals do have only little opportunity, except in the desired way, to control the situation. The question, whether the observable changes in the latency and number of ITS in the MWB are attributable to a differential recruitment of higher fear-regulatory brain areas (e.g., amygdala) and therefore reflect indeed a different behavioral state, cannot be answered currently. But it was shown before that a local re-expression of CB1 within the amygdalar complex (mainly basolateral amygdala and central amygdala in part) in CB1^−/−^ animals was sufficient to restore active fear-coping styles (Metna-Laurent et al., 2012). Taken together our study adds a new facet to our picture about implications of CB1 on glutamatergic vs. GABAergic neurons in controlling escape behavior (i.e., active vs. passive coping strategies; Lutz et al., 2015).

## Author contributions

AJG conceived the idea for the Moving Wall Box, conducted all experiments and wrote the manuscript. CTW conceived the idea for the experiments and commented on the manuscript.

### Conflict of interest statement

The authors declare that the research was conducted in the absence of any commercial or financial relationships that could be construed as a potential conflict of interest.

## Footnotes

^1^https://www.arduino.cc/en/main/software

^2^https://github.com/AGenews/MWB
